# 苯达莫司汀联合泊马度胺、地塞米松治疗伴髓外病变的复发多发性骨髓瘤：多中心研究

**DOI:** 10.3760/cma.j.issn.0253-2727.2023.08.009

**Published:** 2023-08

**Authors:** 弘英 吴, 霞 周, 晓霞 初, 秀芝 邓, 成录 袁, 学红 冉, 国强 刘, 传波 范, 泓媛 郝, 玉萍 钟

**Affiliations:** 1 康复大学青岛医院（青岛市市立医院）血液科，青岛 266071 Qingdao Hospital, University of Health and Rehabilitation Sciences, Qingdao Municipal Hospital, Qingdao 266071, China; 2 烟台毓璜顶医院血液科，烟台 264099 Yantai Yuhuangding Hospital, Yantai 264099, China; 3 威海市立医院血液科，威海 264299 Weihai Municipal Hospital, Weihai 264299, China; 4 山东大学齐鲁医院血液科，青岛 266035 Qilu Hospital of Shandong University, Qingdao 266035, China; 5 潍坊市人民医院血液科，潍坊 261044 Weifang People's Hospital, Weifang 261044, China; 6 胜利油田中心医院血液科，东营 257099 Shengli Oilfield Center Hospital, Dongying 257099, China; 7 青岛市海慈医疗集团，青岛 266033 Qingdao Haici Medical Group, Qingdao 266033, China

**Keywords:** 多发性骨髓瘤, 盐酸苯达莫司汀, 泊马度胺, 髓外病变, Multiple myeloma, Bendamustine hydrochloride, Pomalidomide, Extramedullary disease

## Abstract

**目的:**

评估苯达莫司汀联合泊马度胺、地塞米松（BPD方案）治疗伴髓外病变的复发多发性骨髓瘤（MM）的疗效和安全性。

**方法:**

本项开放、单臂、多中心前瞻性研究纳入2021年2月至2022年11月在青岛市市立医院等7所医院诊断并接受BPD方案治疗的30例伴髓外病变的复发MM患者，分析其疗效及不良反应。

**结果:**

30例患者中位年龄62（47～72）岁，其中18例（60.0％）为首次复发，首次复发的18例患者的总体反应率（ORR）为100％，其中完全缓解3例（16.7％），非常好的部分缓解（VGPR）10例（55.5％），部分缓解（PR）5例（27.8％）。12例二线以上治疗后复发患者的ORR为50％，其中≥VGPR 0例，PR 6例（50％），疾病稳定3例（25％），疾病进展3例（25％）。所有患者的1年无进展生存率为65.2％（95％*CI* 37.2％～83.1％），1年总生存率为90.0％（95％*CI* 76.2％～95.4％）。常见的3～4级血液学不良反应包括中性粒细胞减少2例（6.7％），血小板减少1例（3.3％），总体不良反应可控。

**结论:**

BPD方案在伴髓外病变的复发MM患者中具有良好的疗效和耐受性。

## Abstract (en)

**Objective:**

To evaluate the efficacy and safety of bendamustine combined with pomalidomide and dexamethasone（BPD regimen）in the treatment of relapsed multiple myeloma（MM）with extramedullary disease.

**Methods:**

This open, single-arm, multicenter prospective cohort study included 30 relapsed MM patients with extramedullary disease diagnosed in seven hospitals including Qingdao Municipal Hospital. The patients were treated with BPD regimen from February 2021 to November 2022. This study analyzed the efficacy and adverse reactions of the BPD regimen.

**Results:**

The median age of the 30 patients was 62（47–72）years, of which 18（60％）had first-time recurrence. The overall response rate（ORR）of the 18 patients with first-time recurrence was 100％, of which three（16.7％）achieved complete remission, 10（55.5％）achieved very good partial remission（VGPR）, and five（27.8％）achieved partial remission（PR）. The ORR of 12 patients with recurrence after second-line or above treatment was 50％, including zero patients with ≥VGPR and six patients（50％）with PR. Three cases（25％）had stable disease, and three cases（25％）had disease progression. The one-year progression free survival rate of all patients was 65.2％（95％ *CI* 37.2％–83.1％）, and the 1-year overall survival rate was 90.0％（95％ *CI* 76.2％–95.4％）. The common grade 3–4 hematology adverse reactions included two cases（6.7％）of neutropenia and one case（3.3％）of thrombocytopenia. The overall adverse reactions are controllable.

**Conclusion:**

The BPD regimen has good efficacy and tolerance in relapsed MM patients with extramedullary disease.

多发性骨髓瘤（MM）伴髓外病变（EMD）又称髓外浆细胞瘤，目前国际上尚无统一标准定义，文献中将患者在骨髓原发灶处出现突出于骨骼外或远离骨髓原发灶的软组织浆细胞瘤定义为EMD[1]。文献报道，其发生率随骨髓瘤的进展略有不同，初诊MM伴EMD的发生率为7％～18％，治疗过程中出现EMD的发生率为6％～20％[2]–[3]。近年来，随着MM患者生存期的延长、检查手段的更新及临床医师的重视，EMD的发生率越来越高。髓外浆细胞瘤是独立于其他高危因素的骨髓瘤不良预后类型。MM合并髓外浆细胞瘤的治疗仍面临很大挑战，因尚无统一指南和共识可供借鉴，亟待开展前瞻性临床研究以探寻最优方案。

国外研究报道，苯达莫司汀联合泊马度胺及地塞米松（BPD方案）治疗复发难治MM（RRMM）的总体反应率（ORR）为57.6％[4]，但目前并没有关于苯达莫司汀联合泊马度胺治疗伴EMD的MM的研究。为探索伴EMD的复发MM患者应用BPD方案的有效性和安全性，开展了此项研究。

## 病例与方法

1. 病例：本项开放、单臂、多中心前瞻性研究纳入2021年2月至2022年11月在青岛市市立医院等7所医院诊断并接受BPD方案治疗的30例伴EMD的复发MM患者（注册号：ChiCTR2100048829）。所有患者髓外浆细胞瘤的诊断均由病理活检证实。患者的美国东部肿瘤协作组（ECOG）评分为0～2分。入选标准：①年龄≥18岁；②根据《中国多发性骨髓瘤诊治指南（2020年修订）》[5]中列出的诊断标准具有可测量病灶；③接受过一线治疗且病情进展，复发时伴EMD。排除标准：①活动性乙型肝炎、丙型肝炎，以及其他获得性、先天性免疫缺陷疾病患者；②具有2级或2级以上末稍神经病变或神经痛；③治疗前存在严重血栓事件；④肝功能不全（ALT和AST≥正常值上限2倍）。

所有患者在治疗前完善的实验室检查包括血常规、生化常规（血清白蛋白、球蛋白、总蛋白、血钙、乳酸脱氢酶、肌酐）、β_2_-微球蛋白、血清蛋白电泳、免疫固定电泳、血清游离轻链、外周血形态学分析、骨髓细胞形态学、骨髓活检、免疫分型、荧光原位杂交（FISH）。辅助检查包括心电图、心脏超声、肺部CT、椎体MRI。遗传学高危定义为：FISH检测到t（4;14）、t（14;16）、t（14;20）、17p−。所有患者治疗前根据影像学评估是否合并EMD，同时测量病灶大小。

2. 治疗：BPD方案的具体用法用量如下：苯达莫司汀，静脉滴注，70 mg/m^2^，第1、2天，最长使用6个周期；泊马度胺，口服，4 mg/d，第1～21天，6个周期后继续应用泊马度胺直至疾病进展或出现不可耐受的不良反应；地塞米松，口服，20 mg/d，第1～4、15～18天，老年虚弱患者可酌情减量。28 d为1个治疗周期。在发生3级和4级中性粒细胞减少或血小板减少时，先调整苯达莫司汀的剂量，每次下调上一剂量水平的25％。如苯达莫司汀剂量调整后不良反应仍未缓解或经判定发生了与泊马度胺相关的3级或4级不良反应时调整泊马度胺剂量，1 mg为一个剂量等级，每次下调一个剂量等级。该研究获得青岛市市立医院伦理委员会审查批准（2021临审字Y第012号）。

接受泊马度胺治疗的患者发生过作为严重不良反应报道的静脉血栓栓塞（VTE），对于存在血栓栓塞事件风险的受试者，在泊马度胺治疗期间每日接受阿司匹林预防性治疗或接受另一种预防药物。在有高风险VTE的受试者中，强烈推荐使用低分子量肝素、肝素（根据说明书上用于深静脉血栓/肺动脉栓塞预防的推荐剂量）或华法林（维持INR为2.0）。主要研究终点为ORR、完全缓解（CR）率，次要研究终点为无进展生存（PFS）期。

3. 疗效评估：诱导治疗4个疗程后进行疗效评估。所有患者的疗效评估均根据国际骨髓瘤工作组（IMWG）制定的疗效判断标准[6]，分为疾病进展（PD）、疾病稳定（SD）、部分缓解（PR）、非常好的PR（VGPR）、CR、严格意义的CR（sCR）。ORR＝sCR率+CR率+VGPR率+PR率。诱导治疗结束后行八色流式细胞术监测微小残留病水平。所有患者治疗后根据影像学评估EMD是否治疗有效，同时测量病灶大小。

4. 不良反应：不良反应的判定及分级根据美国国家癌症研究所不良事件常用术语标准（NCICTCAE）4.0版。

5. 随访：采用查阅患者住院、门诊病历及电话随访，随访截止日期为2022年11月30日，中位随访时间11.8（1～22）个月。

6. 统计学处理：基线和人口统计学特征用描述性分析。计数资料以例数（百分比）表示，计量资料用中位数（范围）表示。符合正态分布的资料采用Student's *t*检验。不符合正态分布的资料采用Mann-Whitney *U*检验。生存分析采用Kaplan-Meier法。

## 结果

1. 临床特征：本研究共纳入30例患者，其中18例（60.0％）为首次复发患者，男10例，女8例，中位年龄62（55～71）岁。12例（40.0％）为二线以上治疗复发患者，男7例，女5例，中位年龄62.5（47～72）岁。细胞遗传学高危患者12例，其中首次复发4例，二线以上治疗复发8例（3例为复发后检测）。骨旁软组织肿物8例（26.7％），非骨旁22例（73.3％），包括胸腔积液、下肢肌肉、腮腺、眼部球后、颌下腺软组织肿块等。24例（80.0％）患者为硼替佐米及来那度胺双重耐药。患者的一般临床资料见表1。

**表1:** 30例伴髓外病变的复发多发性骨髓瘤患者的一般临床资料

临床特征	总体（30例）	首次复发（18例）	二线以上复发（12例）
年龄［岁，*M*（范围）］	62（47～72）	62（55～71）	62.5（47～72）
ECOG评分［例（％）］			
0分	6（13.3）	4（22.3）	2（16.7）
1分	15（50.0）	9（50.0）	6（50.0）
2分	9（36.7）	5（27.7）	4（33.3）
ISS分期［例（％）］			
Ⅰ期	3（10.0）	1（5.6）	2（16.6）
Ⅱ期	5（16.7）	3（16.7）	2（16.6）
Ⅲ期	22（73.3）	14（77.7）	8（66.8）
既往治疗线数［例（％）］			
1	18（60.0）	18（100.0）	0（0）
2	8（26.7）	0（0）	8（66.7）
≥3	4（13.3）	0（0）	4（33.3）
细胞遗传学高危［例（％）］	12（40.0）	4（22.2）	8（66.6）
髓外病变部位			
骨旁	8（26.7）	4（22.2）	4（33.3）
非骨旁	22（73.3）	14（77.8）	8（66.6）

**注** ECOG：美国东部肿瘤协作组

2. 疗效与生存分析：30例患者中1例（3.3％）患者治疗早期死亡，28例（96.7％）完成至少2个疗程的治疗，15例（50.0％）患者完成6个疗程的治疗进入维持治疗，6例（20.0％）患者疾病进展更换其他方案。30例患者的ORR为80.0％，其中3例（10％）获得CR，10例（33.3％）获得VGPR，11例（36.7％）获得PR。首次复发的18例患者ORR为100％，3例（16.7％）CR，10例（55.5％）VGPR，5例（27.8％）PR。12例二线以上治疗复发患者的ORR为50％，≥VGPR率为0，PR 6例（50.0％），SD 3例（25.0％），PD 3例（25.0％）。细胞遗传学高危的12例患者ORR为50.0％。3例（10.0％）患者因疾病进展死亡，6例（20.0％）患者疾病进展或复发，中位PFS和OS时间均未达到，1年PFS率为65.2％（95％*CI* 37.2％～83.1％），1年OS率为90.0％（95％*CI* 76.2％～95.4％）。

4. 安全性：治疗期间血液学不良事件中中性粒细胞减少10例（33.3％），其中3级及以上中性粒细胞减少2例（6.7％）。血小板减少6例（20.0％），3级以上血小板减少1例（3.3％）。贫血3例（10.0％），无3级以上贫血发生。非血液学不良事件中消化道不良反应9例（30.0％），主要包括恶心呕吐4例（13.3％）、便秘3例（10.0％）、腹泻2例（6.7％），均为1～2级。其他不良反应包括发热4例（13.3％）、皮疹2例（6.7％）、静脉血栓1例（3.3％），均为1～2级。

## 讨论

MM合并髓外浆细胞瘤的治疗目前仍面临很大挑战，因尚无统一指南和共识可供借鉴，亟待开展前瞻性临床研究以探寻最优方案。泊马度胺作为第三代免疫调节剂，具有显著的抗骨髓瘤活性，适用于既往接受过至少两种治疗（包括来那度胺及蛋白酶体抑制剂）且疾病进展的MM患者。泊马度胺虽然与来那度胺同属于免疫调节剂，但是其与Cereblon（一种泛素连接酶复合物的基质受体）的亲和力更强，具有不同的底物降解动力学，激活不同的下游通路，从而导致不同的抗肿瘤和免疫刺激特性。临床前研究表明，对于来那度胺耐药的细胞系和动物模型，泊马度胺仍具有活性[7]–[10]。一项泊马度胺联合低剂量地塞米松治疗13例髓外浆细胞瘤患者的研究结果显示，ORR为31％，中位OS期为16个月[11]。苯达莫司汀是一种双功能氮芥衍生物，具有独特的烷化剂和抗代谢双重抗肿瘤作用。近年来苯达莫司汀用于治疗RRMM患者的研究数据逐渐增多。有研究显示，苯达莫司汀联合来那度胺和地塞米松治疗一线复发的MM患者的ORR为88.90％，≥VGPR率为56％，中位PFS期为18.6个月，2年OS率为76％。安全性方面，4级以上中性粒细胞减少发生率为34％，血小板减少发生率为16％[12]。NCCN指南中也推荐含有苯达莫司汀的三联方案用于RRMM患者的治疗。

一项研究应用苯达莫司汀联合泊马度胺和地塞米松治疗中位治疗线数5线的RRMM患者，其中硼替佐米既往使用率100％，来那度胺既往使用率100％且全部耐药，ORR为61％，CR率8％，≥VGPR率16％，中位PFS期9.6个月，中位OS期21.3个月。3级以上不良事件中，中性粒细胞减少的发生率为47％，血小板减少发生率21％，贫血发生率26％[13]。苯达莫司汀联合泊马度胺治疗RRMM患者的疗效确切，患者的缓解率和OS时间提高，不良事件可控。目前并没有关于苯达莫司汀联合泊马度胺治疗骨髓瘤伴EMD的研究。

本研究中90％的患者年龄≤70岁，40％的患者合并高危细胞遗传学异常，多线治疗复发患者的细胞遗传学高危比例高于首次复发患者（66.6％对22.2％），3例患者为多线治疗复发后新增细胞遗传学高危。超过70％的患者为非骨旁软组织肿物。本研究结果显示，首次复发患者的ORR为100％，总体ORR为80％，较既往文献报道的57.6％和61％明显提高[4],[13]，原因可能为本研究中有18例（60％）为首次复发的MM患者，而既往文献应用BPD方案的MM患者中位治疗线数为5（3～8）线[13]。另外，我们的病例中首次复发患者的细胞遗传学高危比例低于多线治疗复发患者，也是本研究疗效更好的原因之一，提示尽早应用本方案可提高疗效。

本项研究均为高危患者，肿瘤负荷较高，所有患者既往接受过来那度胺或硼替佐米治疗后复发，结果显示，BPD方案在具有髓外浆细胞瘤的MM患者中获得了良好的缓解率和生存结局。该方案安全性较好，不良反应可控，在本研究中未观察到新的不良事件。最常见的不良反应为血液学不良反应，其发生频率和严重程度与泊马度胺-地塞米松MM-003试验中观察到的不良反应相似，与其他基于泊马度胺、地塞米松的三药方案或基于苯达莫司汀的三药方案相比也无差异[14]–[15]。大多数血液学不良反应出现于第1个治疗周期，通过降低剂量和应用粒细胞集落刺激因子支持可以控制。在应用BPD方案时，监测全血细胞计数（尤其是第1个疗程的全血细胞计数）是有帮助的，仅有3例患者出现≥3级血液学不良反应。

我们的研究有一定的局限性，如作为一项多中心研究者发起的临床试验样本量不够大，细胞遗传学数据缺乏等。总之，BPD方案用于伴EMD的复发MM患者具有较好的疗效和可接受的不良反应，后续需要扩大样本量进一步研究。
